# Spermidine coupled with exercise rescues skeletal muscle atrophy from D-gal-induced aging rats through enhanced autophagy and reduced apoptosis via AMPK-FOXO3a signal pathway

**DOI:** 10.18632/oncotarget.15728

**Published:** 2017-02-25

**Authors:** Jingjing Fan, Xiaoqi Yang, Jie Li, Ziyang Shu, Jun Dai, Xingran Liu, Biao Li, Shaohui Jia, Xianjuan Kou, Yi Yang, Ning Chen

**Affiliations:** ^1^ Hubei Key Laboratory of Exercise Training and Monitoring, Hubei Provincial Collaborative Innovation Center for Exercise and Health Promotion, College of Health Science, Wuhan Sports University, Wuhan, China; ^2^ Graduate School, Wuhan Sports University, Wuhan, China; ^3^ Graduate School, Jilin Sport University, Changchun, China

**Keywords:** D-galactose, spermindine, exercise, autophagy, AMPK-FOXO3a signal pathway, Gerotarget

## Abstract

The quality control of skeletal muscle is a continuous requirement throughout the lifetime, although its functions and quality present as a declining trend during aging process. Dysfunctional or deficient autophagy and excessive apoptosis may contribute to the atrophy of senescent skeletal muscle. Spermidine, as a natural polyamine, can be involved in important cellular functions for lifespan extension and stress resistance in several model organisms through activating autophagy. Similarly, cellular autophagic responses to exercise have also been extensively investigated. In the present study, in order to confirm the mitigation or amelioration of skeletal muscle atrophy in aging rats through spermidine coupled with exercise intervention and explore corresponding mechanisms, the rat model with aging-related atrophy of skeletal muscle was established by intraperitoneal injection of D-galactose (D-gal) (200 mg/kgd), and model rats were subjected to the intervention with spermidine (5 mg/kgd) or swimming (60 min/d, 5 d/wk) or combination for 42 days. Spermidine coupled with exercise could attenuate D-gal-induced aging-related atrophy of skeletal muscle through induced autophagy and reduced apoptosis with characteristics of more autophagosomes, activated mitophagy, enhanced mitochondrial quality, alleviated cell shrinkage, and less swollen mitochondria under transmission scanning microscopic observation. Meanwhile, spermidine coupled with exercise could induce autophagy through activating AMPK-FOXO3a signal pathway with characterization of increased Beclin1 and LC3-II/LC3-I ratio, up-regulated anti-apoptotic Bcl-2, down-regulated pro-apoptotic Bax and caspase-3, as well as activated AMPK and FOXO3a. Therefore, spermidine combined with exercise can execute the prevention or treatment of D-gal-induced aging-related skeletal muscle atrophy through enhanced autophagy and reduced apoptosis mediated by AMPK-FOXO3a signal pathway.

## INTRODUCTION

Skeletal muscle mass represents roughly 40-50% of human body weight as the largest tissue and the major amino acid reservoir, and may vary according to physiological and pathological conditions [1]. One of the major tissues affected during aging process is skeletal muscle, which is characteristics of decreased mass and fiber size [2]. The atrophy of skeletal muscle is a highly prevalent and disabling condition as the rapid increase in the number of aged people, but knowledge about cellular signal pathways mediating muscle atrophy is still limited. During the aging process with gradual loss of capability to adapt changing environments, the decreased regeneration capacity and negative protein turnover as well as mitochondrial dysfunction of skeletal muscle are usually considered to be the molecular mechanisms involving in the pathogenesis of aging-related atrophy of skeletal muscle [2]. According to previous studies, the animals subjected to the treatment of D-galactose (D-gal) have shown several aging hallmarks, such as reduced longevity, increased oxidative stress [3], decreased activity of antioxidant enzymes [4], increased mitochondrial DNA mutation [5] and mitochondrial dysfunction [6], which may be also correlated with skeletal muscle atrophy associated with aging.

A number of critical signaling pathways correlated with aging-related hallmarks can further promote the aging process. It is worthy of attention that aging-related myopathy usually presents an exacerbation of apoptosis and an abnormal functional status of autophagy including deficient autophagy or dysfunctional autophagic flux due to the accumulation of damaged products and overall decrease of protein degradation process [7]. The signaling pathways of autophagy and apoptosis in mammalian cells are sensitive to intracellular homeostasis and can be either activated or deactivated by exoteric stress, such as toxins, insufficient or excessive intake of nutrients and exercise [8]. With the extension of age, the accumulation of metabolic wastes and damaged cells or organelles may disrupt the effective regulation of autophagy and apoptosis. In turn, a declined or deficient autophagy as well as dysfunctional autophagic flux during aging process could cause more accumulation of dysfunctional organelles and unfolded proteins [9, 10], which stimulates us to focus on exercise and drug intervention strategies such as caloric restriction mimetics and antioxidants for the prevention and/or amelioration of aging-associated skeletal muscle atrophy.

Regular and appropriate exercise is a common intervention and a well-known stimulus for muscle adaptation and energy metabolism of mitochondria in skeletal muscle, which is often used to attenuate the loss of skeletal muscle in aged population. During the intervention using appropriate exercise, both autophagy and apoptosis could be induced in skeletal muscle to limit tissue damage, restore tissue integrity, terminate inflammatory responses, or induce direct signal pathways for adaptation [11–13]. Currently, the attenuation of basal autophagy with the extension of age is confirmed, and endurance exercise training has been shown to enhance autophagic signaling in aged mice [14]. Moreover, regular exercise has been demonstrated to alleviate apoptosis and DNA fragmentation in skeletal muscle through down-regulating apoptotic proteins such as Bax and caspases, and up-regulating apoptotic inhibitors such as Bcl-2. However, the regulatory mechanisms responsible for the activation or the balance of autophagy and apoptosis in aging skeletal muscle during different types of exercise training are still debatable.

Spermidine has been identified as a potent and specific inducer of autophagy for expending lifespan due to the hypoacetylation of histone, which can provide protection for aging process of several tissues including heart, brain and skeletal muscle, thereby accomplishing the longevity [15–19]. A recent study has documented that, intracellular concentration of spermidine, with a declining trend during human aging process, is connected with the lifespan of yeast, flies and worms, and human cells due to the deficient autophagy [20]. Spermidine can not affect the phosphorylation of mTOR and its substrate ribosomal protein S6 kinase, but can induce autophagy through AMP-activated protein kinase (AMPK)-dependent signal pathway [21]. Moreover, spermidine has the function to induce autophagy in most eukaryotes and reduce oxidative stress. Hence, spermidine may decrease endoplasmic reticulum (ER) stress and reduce apoptosis *via* activating AMPK-dependent autophagy pathway [22].

Excessive apoptosis and deficient autophagy may cause the atrophy of skeletal muscle under pathological events or aging process, and correspondingly may promote cell death and disease progression [2]. Therefore, the functional status of autophagy and apoptosis should be the determinant for maintaining long-term health of skeletal muscle [23]. AMPK has been confirmed to regulate protein metabolism and many cellular processes that are disrupted in skeletal muscle of aging subjects [24]. The activation of AMPK also can slow down the process of senescence by regulating different signal pathways [25], and has been investigated as a therapeutic target in aging-related myopathies [26]. FOXO3a, as a downstream effector of AMPK signaling, is known to be associated with longevity [27]. The up-regulation of FOXO3a can attenuate the expression of atrophy-related proteins in skeletal muscle [28]. Therefore, we hypothesized that long-term regular exercise combined with a specific autophagic inducer spermidine could activate autophagy and inhibit apoptosis to establish this “just right” level for health maintenance of skeletal muscle. To test this hypothesis, we attempted to demonstrate the relationship between exercise and autophagy *in vivo* using a rat model, subjected to the intervention of aerobic swimming combined with an anti-aging drug, spermidine, to confirm whether enhanced autophagy and reduced apoptosis could contribute to the mass maintenance of skeletal muscle in aging-related muscle atrophy, thus implying the critical regulatory role of FOXO3a and AMPK signaling in skeletal muscle of D-gal-induced aging model rats with deficient autophagy and excessive apoptosis.

## RESULTS

### Spermidine and exercise reduced D-gal-induced aging and damage of skeletal muscle

Aging is known to be associated with a slow decline in skeletal muscle functions accompanied by progressive loss in the mass and strength of skeletal muscle. A decrease in skeletal muscle mass is a reliable indicator of aging process. In the present study, in order to understand the loss of skeletal muscle upon D-gal exposure, we examined the ratio of gastrocnemius muscle weight to body weight (GWM/BW ratio). The GWM/BM ratio of D-gal-treated rats exhibited a significant decline when compared with that in rats from Con group (ANOVA, *n* = 7; *P* < 0.001), suggesting that D-gal administration can induce the mass loss of skeletal muscle in rats. In contrast, spermidine or exercise (DS or DE group) could protect the impairment slightly with less body weight and a little increase in muscle mass. This could be due to the type of our exercise training, swimming. Endurance training could not only delay the process of aging-related muscle loss, but can be benefit for cardiopulmonary function [29], insulin sensitivity [30], and mitochondrial quality control [31], while it could offer less muscle mass than strength development [32]. Although the difference of GWM/BM ratio between individual intervention group (DS or DE group) and D-gal group did not exhibit a statistical significance, the intervention of spermidine coupled with exercise (DES group) could result in an obvious protection for D-gal-induced damage of skeletal muscle (Table 1).

**Table 1:** Spermidine coupled with exercise alleviated the loss or atrophy of skeletal muscle in D-gal-induced model rats with aging (*n* = 7)

Groups	Body weight (g)	Gastrocnemius muscle (g)	GMW/BW ratio (×100)
D	402.6 ± 20.488	4.834 ± 0.241	1.201 ± 0.011***
DS	398.6 ± 16.618	4.861 ± 0.211	1.220 ± 0.016
DE	381.8 ± 23.984	4.589 ± 0.241	1.205 ± 0.018
DES	373.2 ± 9.281	4.632 ± 0.119	1.241 ± 0.008#
Con	430.0 ± 28.244	5.556 ± 0.342	1.294 ± 0.012

Data were presented as mean ± standard error (M ± SE). *P < 0.05, ***P < 0.001 when D-gal group compared with the normal control group. #*P* < 0.05 when DES group compared with the D-gal model group.

**Figure 1:**
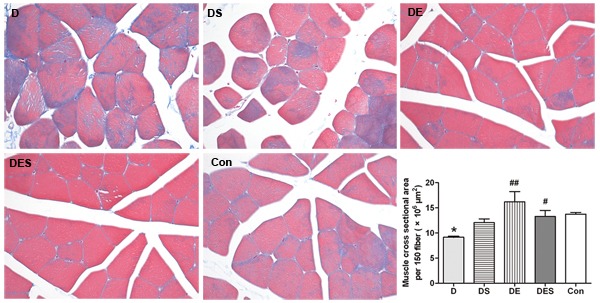
Spermidine coupled with exercise training reduced D-gal-induced damage of skeletal muscle, and suppressed or rescued the decline of sectional area of skeletal muscle fibers Cross sectional area was measured and calculated from 150 skeletal muscle fibers in H&E stained sections using imageJ software. All rats were divided into five groups (*n* = 7) including rats treated with saline (Con), rats treated with D-gal (D), rats treated with D-gal coupled with spermidine (DS), rats treated with D-gal coupled with exercise training (DE), and rats treated with D-gal and spermidine as well as exercise training (DES) (×200).

As shown in Figure 1, in order to evaluate whether there are any morphological changes associated with D-gal-induced aging process, we performed masson trichrome staining to characterize the internal structure of skeletal muscle fibers and to examine the morphology of gastrocnemius muscle with or without exercise training and spermidine intervention. The cross-sectional areas of gastrocnemius muscle were qualitatively and quantitatively analyzed under a light microscope. Morphological changes were observed in three randomly selected fields with masson staining. Under normal conditions, myofilaments revealed an orderly and neat arrangement. All nuclei were located at the edge of skeletal muscle fibers, whereas skeletal muscle fibers in D-gal-induced aging group had an obvious damage with loose structure and disordered arrangement. After spermidine and exercise training intervention for 6 weeks, the disordered structure of myofilaments was obviously improved. These results suggest that D-gal-induced aging might accelerate the atrophy of skeletal muscle, and could be alleviated by regular exercise training. Meanwhile, histochemistry staining also showed a trace of local collagen fibrils (connective tissue) among skeletal muscle in each group. As one of the important characteristic hallmarks for the aging of skeletal muscle, the fibrosis in gastrocnemius samples of the rats receiving D-gal injection was more severe when compared to the young normal control rats, which was alleviated after daily intervention with spermidine and exercise (Figure 1). At the same time, we also evaluated the cross-sectional area of skeletal muscle fibers between groups. As shown in Figure 1, a corresponding reduction in cross-sectional areas was observed in D-gal-induced aging rats when compared to young normal control rats (*P* < 0.05). However, a significant increase in cross-sectional areas of skeletal muscle fibers in rats from DE (*P* < 0.001) and DES (*P* < 0.01) groups was detected.

**Figure 2:**
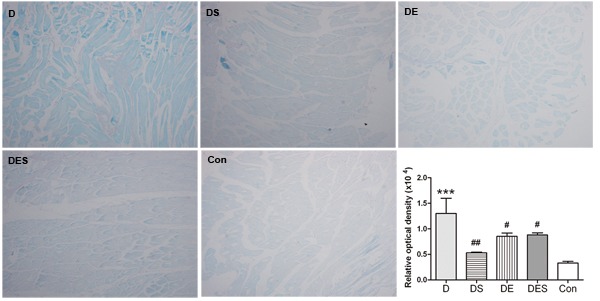
Effects of spermidine and/or exercise on the damage of gastrocnemius muscle in D-gal-induced aging rats through the evaluation of SA-β-gal staining (×40) The positive cells with SA-β-gal staining (blue) due to D-gal-induced senescence exhibited a decrease in spermidine and exercise groups when compared with D-gal-treated group.

We have speculated that the loss of skeletal muscle function in rats from D-gal administration group is similar with those from sarcopenia. As one of the most widely used biomarkers for aging cells, the increased β-galactosidase during senescence has been reported and used widely as a biomarker of cellular senescence. As shown in Figure 2, SA-β-gal positive cells with blue staining were rarely observed in Con and DES groups. The D-gal-induced aging was evaluated by relative optical density (ROD) for positive cells with SA-β-gal blue staining. The ROD of positive cells did not reveal statistically significant difference between Con group and DES group. Remarkably, D-gal administration induced a remarkable increase in SA-β-gal staining when compared with Con group (Figure 2). However, DE, DS and DES groups could result in reduced ROD, suggesting that these treatments could attenuate or protect D-gal-induced senescence of skeletal muscle.

**Figure 3:**
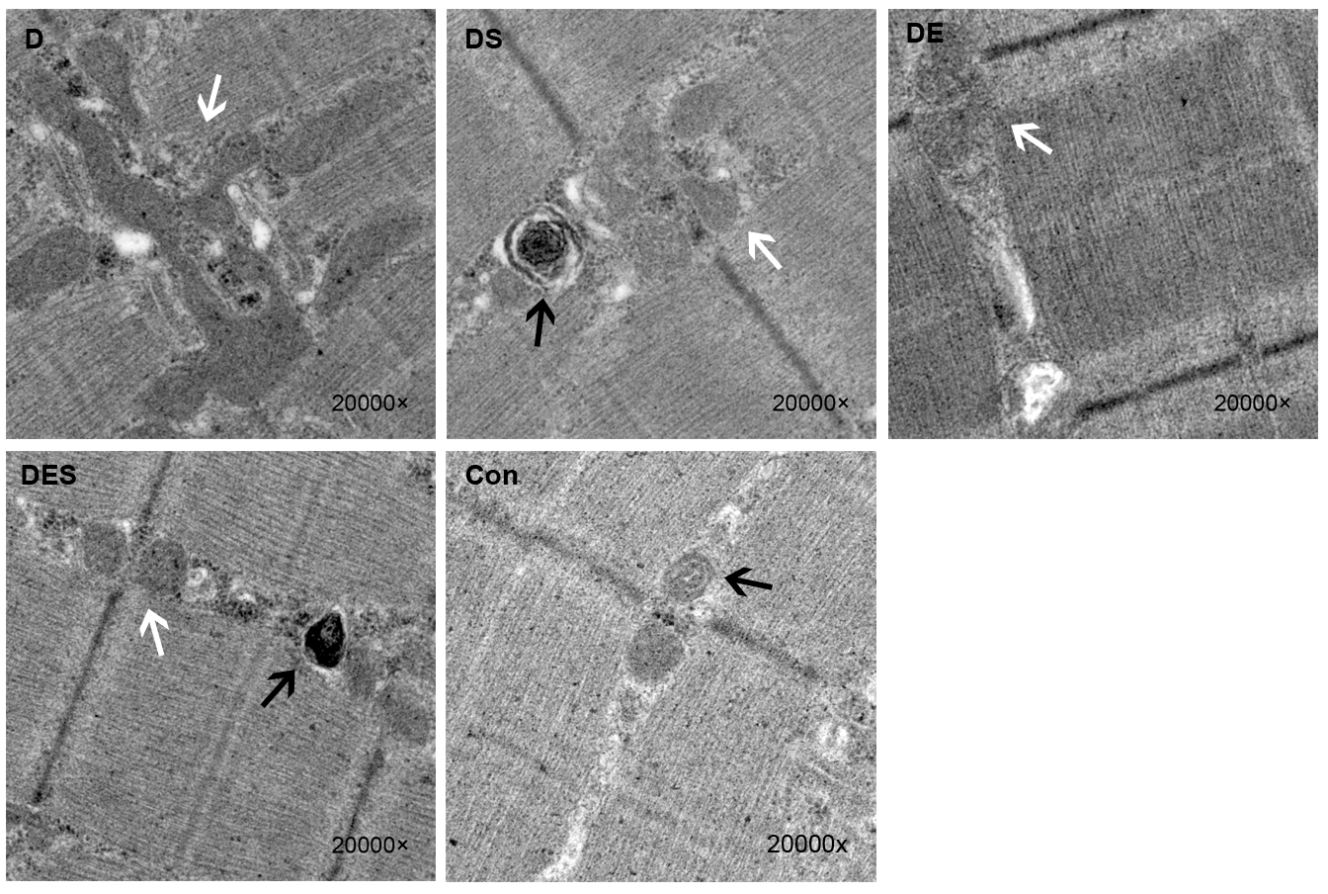
Transmission electron microscopic images showed damaged or swollen or fusion mitochondria, and disordered fiber arrangement or dysfunctional fiber structure in D-gal-induced aging gastrocnemius muscle (×20000) Spermidine and/or exercise improved the disordered fiber arrangement and reduced the population of damaged or fusion mitochondria of D-gal-induced aging skeletal muscle fibers. White arrows represent the region of mitochondria, and black arrows point to the autophagosomes in gastrocnemius muscle.

In addition, the formation of autophagosomes and the quality or functional status of mitochondria in skeletal muscle were examined and evaluated by transmission electron microscope. Compared with the normal control group, many vacuoles with different sizes, and subsarcolemmal accumulation of enlarged and abnormally shaped or swollen mitochondria were observed in D-gal-induced aging group (Figure 3). One of the hallmarks for the aging of skeletal muscle is the high population of damaged or swollen mitochondria, and disordered fiber arrangement or dysfunctional fiber structure in skeletal muscle. The normal arrangement of mitochondria at the I band on either side of the Z disc and apparently more autophagosomes were observed in skeletal muscle from DS and DES groups, which was rarely observed in skeletal muscle from D-gal-induced aging model group (Figure 3).

### Spermidine combined with exercise attenuated oxidative stress in D-gal-induced aging rats

**Figure 4:**
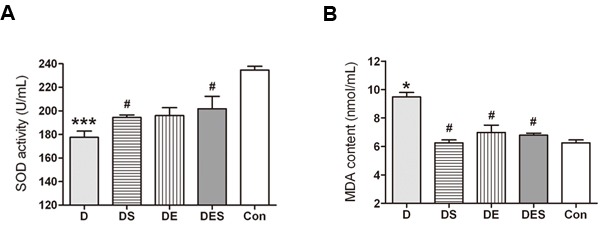
Effects of spermidine and exercise on SOD activity and MDA content of aging model rats (*n* = 5) *P < 0.05, ***P < 0.001 when compared with the normal control group. ^#^*P* < 0.05 when compared with the D-gal group.

To understand the underlying mechanisms for the protective roles of spermidine and exercise, we measured senescence-associated biomarkers such as SOD activity and MDA content in serum of the rats from each group. As shown in Figure 4, SOD activity in serum from D-gal-induced aging model rats (177.54 ± 9.12 U/mL) significantly declined when compared with that in the normal control group (234.67 ± 5.47 U/mg protein) (*P* < 0.001), whereas the decrease was significantly attenuated in the presence of spermidine at the daily dose of 5 mg/kg (194.46 ± 3.6 U/mL, *P* < 0.05). The SOD activity from exercise or spermidine alone group also revealed an increasing trend (196.06 ± 11.55 U/mL; 201.83 ± 18.16 U/mL, respectively). There was a significant increase (9.479 ± 0.4419 nmol/mL; *P* < 0.05) in MDA level in D-gal-induced aging model group (D) when compared with the young normal control group (Con) (6.246 ± 0.2783 nmol/mL; *P* < 0.05); however, the MDA level in SPD intervention groups (DS and DES) exhibited a significant reduction (6.250 ± 0.2946 and 6.791 ± 0.2110 nmol/mL, respectively; *P* < 0.05). The observed reduction is similar to the results obtained from DE group (6.979 ± 0.7366 nmol/mL; *P* < 0.05).

### Up-regulation of critical regulators by exercise and spermidine could result in the activation of autophagy in D-gal-induced aging skeletal muscle

**Figure 5:**
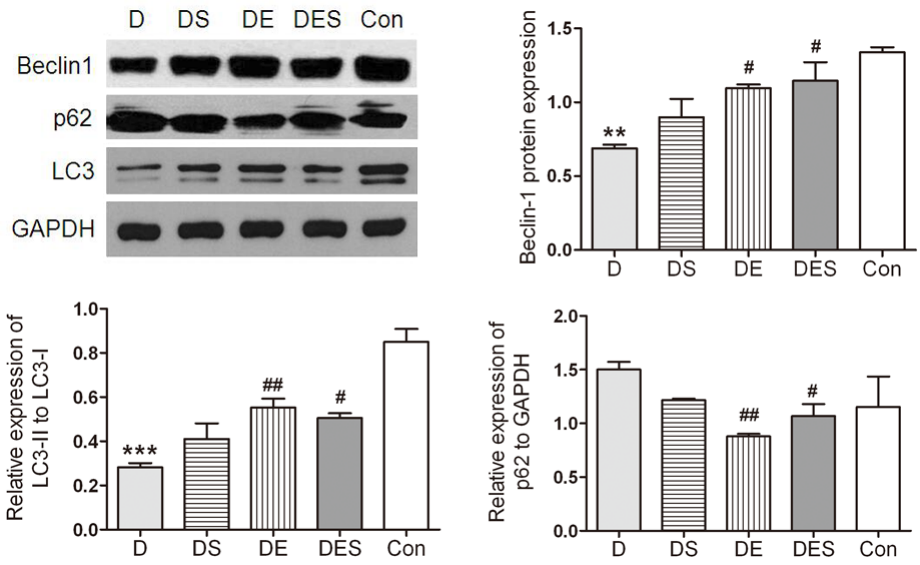
Spermidine and exercise resulted in the activation of autophagy in skeletal muscle and the recovered autophagy during the D-gal-induced atrophy process of skeletal muscle due to up-regulated Beclin-1 and down-regulated p62 as well as increased LC3-II/LC3-I ratio GAPDH was used as the loading control. *P < 0.05, **P < 0.01 when compared with the control group; ^#^*P* < 0.05 when compared with the D-gal group.

In order to evaluate whether exercise and spermidine can rescue the deficient autophagic signals induced by D-gal administration, the functional status of autophagy after spermidine injection and exercise training was determined. The gastrocnemius muscle samples were harvested for Western blotting analysis. The biomarkers associated with autophagy such as Beclin1, microtubule-associated protein light chain 3 (LC3), and p62 were determined. As shown in Figure 5, the ratio of LC3-II to LC3-I was significant lower because D-gal administration can lead to an accelerated aging process, and dysfunctional autophagy. In the present study, Western blotting analysis revealed the increased expression of Beclin1 in DE and DES groups when compared with that in D-gal-induced aging model group (*P* < 0.01); both DE and DS groups revealed a considerable increase in LC3-II/LC3-I ratio (*P* < 0.05) when compared with the rats with D-gal treatment. Furthermore, the content of p62 exhibited the highest level in the D-gal-induced aging model group (*P* < 0.05) than in other groups, suggesting lower autophagic flux in D-gal-induced aging skeletal muscle. In contrast, swimming intervention group showed a significantly decreased expression of p62 when compared with D-gal model aging group (*P* < 0.05).

### Exercise and spermidine could result in the reduction of excessive apoptosis in D-gal-induced aging skeletal muscle

**Figure 6:**
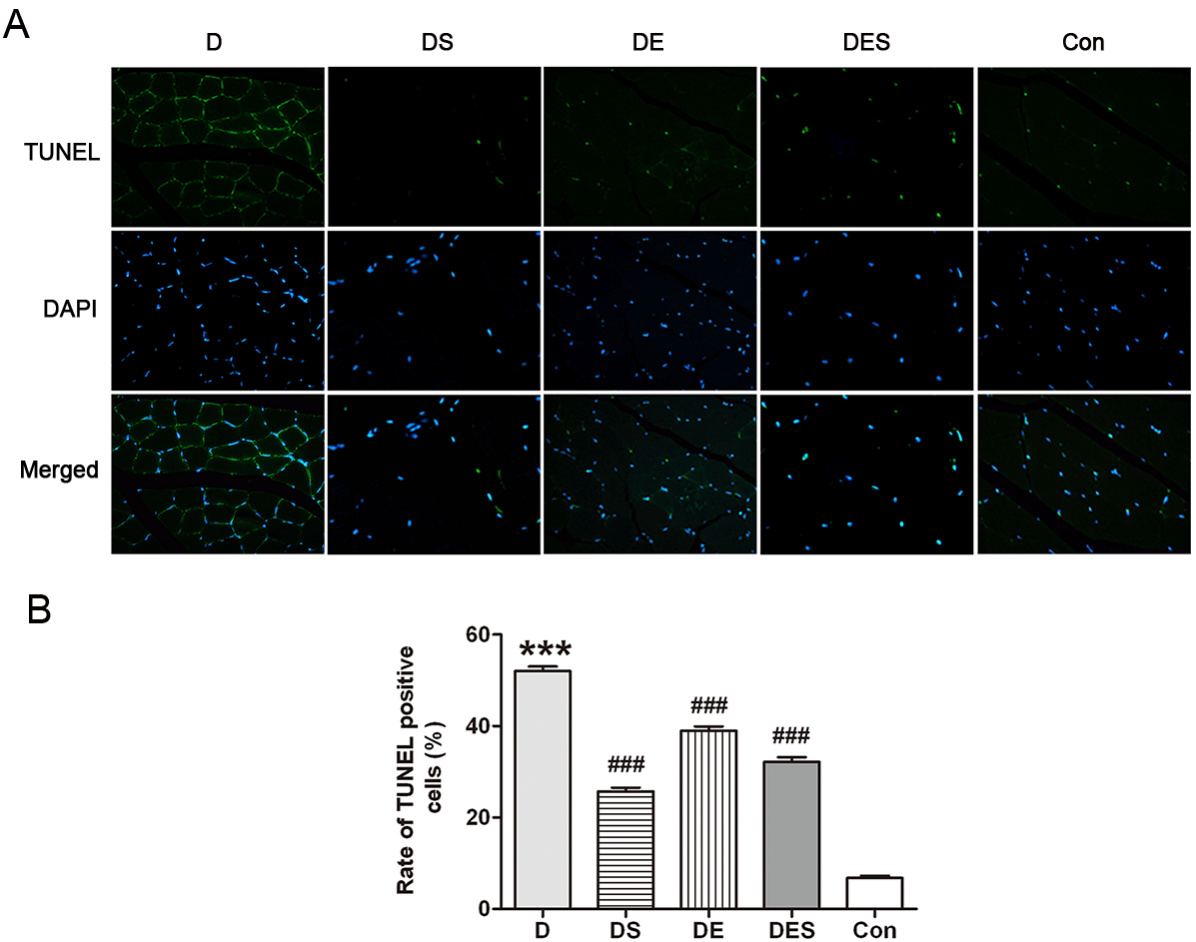
Apoptosis of skeletal muscle cells in different groups was evaluated by TUNEL assay, and the images were recorded by an Olympus fluorescence microscope, captured with a CCD monochrome camera Sensystem1401E at the magnification of ×800 ***P < 0.001 when compared with the control group; ^###^*P* < 0.001 when compared with the D-gal group.

Since autophagic protein levels were increased in exercise and spermidine groups, we next analyzed whether apoptotic signal pathways were affected during exercise and spermidine intervention. In order to investigate the protective effect of spermidine and exercise on aging skeletal muscle cells, D-gal-induced aging rats were pretreated with spermidine and swimming training. Usually, apoptosis occurs mainly in capillary endothelial cells in aging skeletal muscle [33], while we found that the skeletal muscle of the rats from D-gal-induced aging group exhibited a significantly higher proportion of apoptotic cells when compared with the normal control group, as shown in the TUNEL analysis (Figure 6A). Moreover, the rate of TUNEL-positive cells was significantly reduced after spermidine and exercise interventions. The population of apoptotic cells induced by D-gal significantly decreased after pretreatment with spermidine. As shown in Figure 6B, D-gal administration caused an obvious increase in apoptosis (52.02% *versus* 6.77%, *P* < 0.001) when compared to the normal control group. Either spermidine or exercise could significantly alleviate D-gal-induced apoptosis (25.74/38.97 *versus* 52.02%, *P* < 0.001), but no apparent difference was observed among spermidine, exercise and combinatorial intervention groups.

**Figure 7:**
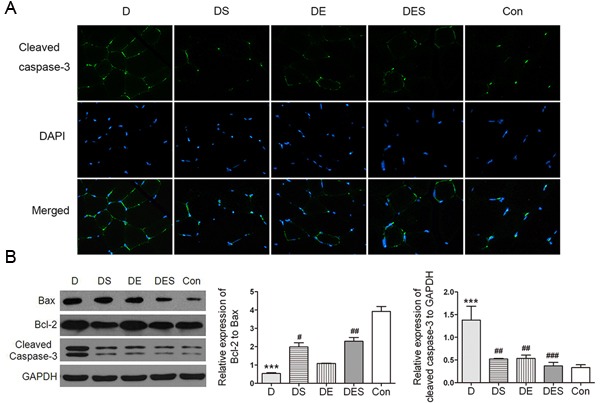
Spermidine combined with exercise could reduce apoptosis in D-gal-induced atrophy of gastrocnemius muscle **A**. Immunofluorescence assay was used to localize the expression of cleaved caspase-3 (green). Nuclei were marked with DAPI (blue), magnification (×800). **B**. Expression of apoptosis-associated proteins and quantitative analysis for Western blotting. GAPDH was used as the loading control. Data were expressed as mean ± standard error (M ± SE). **P < 0.01, ***P < 0.001 when compared with the control group; #*P* < 0.05, ##*P* < 0.01, ###*P* < 0.001 when compared with the D-gal group.

To investigate the pro-apoptotic activity, Western blotting analysis for Bax, Bcl-2 and cleaved caspase-3 as central modulators of intrinsic apoptosis was performed in skeletal muscle of the rats from each group after D-gal treatment. The apoptotic marker such as cleaved caspase-3 was significantly reduced after SPD and exercise intervention when compared to D-gal-induced aging model group (Figure 7A). Meanwhile, a significant enhanced Bcl-2/Bax ratio was observed in DES group. However, it seems that only spermidine has major effect while exercise has a minor effort compared with spermidine (Figure 7B). These observations indicate that the protective mechanism in response to spermidine and exercise in D-gal-induced aging model rats is to adequately suppress apoptotic signal pathways.

### Spermidine combined with exercise increased autophagy and attenuated apoptosis by activating AMPK-FOXO3a signal pathway

**Figure 8:**
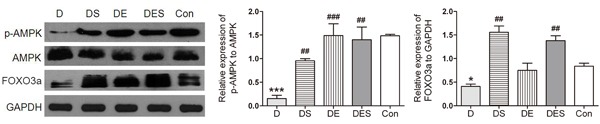
Spermidine combined with exercise enhanced AMPK-FOXO3a signal pathway in D-gal-induced aging gastrocnemius muscle **A**. Protein expression of p-AMPK (Thr72)/AMPKα (D5A2) and FOXO3a. **B**. Quantitative analysis for Western blotting. GAPDH was used as the loading control. Data were expressed as mean ± standard error (M ± SE). *P < 0.05, ***P < 0.001 when compared with the control group; #*P* < 0.05, ##*P* < 0.01, ###*P* < 0.001 when compared with the D-gal group.

Autophagy can be enhanced by the activated AMPK signal pathway [34]. The p-AMPK/AMPK ratio was detected to confirm this mechanism with the activation of autophagy. In keeping with this notion, the results suggest that FOXO3a is involved in spermidine and exercise actions on the prevention of senescence. The D-gal treatment attenuated this alteration, and the phosphorylation of AMPK and the expression of FOXO3a in D group revealed the reduction by 89.7% and 51%, respectively, when compared with the Con group (*P* < 0.001), whereas spermidine and exercise intervention could increase the ratio of p-AMPKα (Thr72) to AMPKα (D5A2) (Figure 8). Western blotting analysis showed the significantly up-regulated expression of p-AMPKα (Thr72) in spermidine and combinatorial groups. Similar results were achieved in the expression of FOXO3a. Therefore, our results confirmed that spermidine and exercise could rescue the deficiency or dysfunction of autophagy in skeletal muscle cells in D-gal-induced aging rats by activating AMPK-FOXO3a signal pathway (Figure 8)

## DISCUSSION

The prevalence of sarcopenia reached up to 33% in community-dwelling elderly, and this number is expected to increase with the rapid expansion of elderly and obese population [35]. Although multiple factors such as short telomere defect, myosin heavy chain isozyme switch, mitochondrial damage, oxidative stress, and impaired intracellular calcium signaling are characterized for the aging process of skeletal muscle, the precise underlying mechanisms for the aging of skeletal muscle remains elusive. Aging-related skeletal muscle atrophy is known not only to accelerate the loss of skeletal muscle fiber and strength, but also to be associated with mobility impairment, an increased risk of falls, and physical frailty [36]. The analysis for the impact of aging on the internal structure of skeletal muscle fibers indicates that Type II fiber atrophy is an early contributor to aging-related muscle atrophy [37]. Therefore, gastrocnemius muscle is chosen as the object in the present study.

As an inducer of aging *in vivo*, D-gal is extensively used for exploring the targets of aging modeling and drug testing, and many experimental animal models with D-gal-induced acute aging and tissue damage have already developed in previous studies [38–40]. Therefore, in the present study, we applied D-gal-induced aging rat model to simulate aging-related damage of skeletal muscle. D-gal-treated rats showed typical aging symptoms, as evidenced by increased senescence-associated β-galactosidase and cell damage, which is consistent with previous demonstration that D-gal can cause the accumulation of reactive oxygen species (ROS) with a final oxidative stress [39]. Although the optimal dose and duration of D-gal administration are required to produce an aging rat model, the effect and toxicity of D-gal (150, 200 and 300 mg/kg) and SPD (1, 5 and 10 mg/kg) at various time durations have been systematically evaluated in our pilot experiments (data not shown). Therefore, the daily concentrations of D-gal and SPD with 6 weeks intervention is optimized to be 200 mg/kg and 5 mg/kg based on our preliminary experiments, which not only can induce the sub-acute aging model, but also can result in less drug toxicity. In the present study, the SA-β-gal staining and TEM assay showed that D-gal treatment could result in the aging of skeletal muscle in rats (Figure 3). Similarly, increased MDA content and reduced SOD activity was observed in serum of D-gal-induced aging rats, indicating that the animal model of mimetic aging by D-gal administration was successfully established. According to the mitochondrial-lysosomal theory, autophagy, or even mitophagy, reveals an obvious decline during aging process in long-lived post-mitotic tissues such as skeletal muscle, thus leading to an accumulation of dysfunctional and enlarged “giant” mitochondria [41]. The significant enlargement with the increased branching and length of mitochondria reported in aged skeletal muscle [42] is also consistent with our finding (Figure 3), which further supports the mitochondrial-lysosomal theory for the homeostasis and mitochondrial quality control in skeletal muscle. It is well known that mitochondrial fusion is important for the maintenance of mitochondrial function and integrity; therefore, the change in mitochondrial morphology observed here might be an increase in fusion to compensate for impaired mitophagy.

The purpose of our study is to evaluate the protective effects of spermidine and exercise on skeletal muscle mass in sub-acute aging model induced by D-gal and to explore corresponding mechanisms. We also investigated the signal pathways for the regulation of autophagic and apoptotic signals in the presence of spermidine and exercise. As a ubiquitin-like protein conjugated to PE, LC3 is involved in cargo recruitment and autophagosome biogenesis [43]. During the formation of autophagosomes, LC3-I is processed to LC3-II by lipidation. Lipidation of LC3 results in an increased LC3-II/LC3-I ratio as a golden marker of autophagy induction in different tissues including skeletal muscle. Thus, the level of LC3-II reveals an increase during the generation of autophagosomes [44, 45]. A decline in autophagic levels has been described in skeletal muscle of aging animals. The qualitative or quantitative monitoring of LC3 is also applied for evaluating autophagic flux *via* LC3-II/LC3-I ratio coupled with the degradation status of p62. In aged animals, chronic exercise training can significantly reduce DNA fragmentation, cleaved caspase-3 content, Bax level, and Bax/Bcl-2 ratio [46]. Correspondingly, these data support the notion that regular exercise may be beneficial to skeletal muscle by decreasing DNA fragmentation and promoting an anti-apoptotic environment. Our results have also confirmed that regular exercise combined with spermidine could ameliorate skeletal muscle atrophy and excessive apoptosis in D-gal-induced aging skeletal muscle with deficient autophagy through comprehensively evaluating above indicators associated with autophagy and apoptosis, and achieving the consistent results.

There are enough publications as the evidence to support the fact that the activation of autophagy can reduce or inhibit apoptosis, and vice versa [47, 48]. The genetic inhibition of autophagy by knockout or knockdown of ATG genes often leads to apoptotic or necrotic cell death [49–51], which clearly reveals that the inhibition of autophagy contributes to the activation or prevalence of apoptosis. Bcl-2 is an anti-apoptotic protein acting as an indirect negative regulator of autophagy by binding and preventing Beclin1 from inducing autophagy [52]. Conversely, the Bcl-2 family member, Bax, is pro-apoptotic, and also can suppress autophagy through caspase-mediated cleavage of Beclin1, which prevents Beclin1 from initiating autophagy [53]. The interaction between the anti-apoptotic protein Bcl-2 and the autophagy protein Beclin1 is essential to regulate the switch between autophagy and apoptosis. Bcl-2 binds to Beclin1 and segregates Beclin1 away from class III PI3K, thereby leading to an inhibition of autophagic response. This suggests that apoptosis activation results in autophagy suppression in a context-dependent manner. In contrast, the activation of autophagy could inhibit apoptosis by exercise or autophagic activator [54, 55].

As a key regulator of mitochondrial biogenesis, AMPK inhibits mTOR in response to reduced ATP levels. AMPK suppresses protein synthesis by down-regulating mTOR signaling cascade, which regulates numerous components involved in protein synthesis, including initiation and elongation factors, and the biogenesis of ribosomes themselves. AMPK is an energy sensor that also activates autophagy and inhibits apoptosis [56]. As far as we know, a number of reports indicate that AMPK activity is correlated with the atrophy of skeletal muscle [57]. Further elucidation of the role of regular exercise combined with spermidine in protecting aging cells and decreasing cell death will improve the intervention effectiveness. Both regular exercise and spermidine have been demonstrated to activate the phosphorylation of AMPK. FOXO proteins (FOXO1, FOXO3a, FOXO4 and FOXO6) are an evolutionarily conserved subfamily of transcription factors involved in a variety of cellular processes with a diversity of functions including controlling cell cycle and differentiation, resisting oxidative stress and apoptosis in response to stress stimuli in human cells [58, 59]. A large volume of evidence indicates that both FOXO1 and FOXO3 have the regulatory roles in various models with skeletal muscle atrophy. The overexpression of either FOXO1 or FOXO3 can produce skeletal muscle atrophy *in vivo* [60, 61]. FOXO1 has been shown to regulate the differentiation of myotubes and the remodeling of skeletal muscle fiber types, thereby leading to the expression of Atrogin-1 and MuRF-1 proteins that are important to the development of skeletal muscle atrophy [62, 63]. FOXO3 can execute its function by stimulating overall protein degradation and coordinately activating both UPS and autophagy signal pathways in atrophying muscle cells [64]. However, UPS may have the less function on the aging-related muscular atrophy due to no significant aging-dependent change in the expression of atrogin-1 and MuRF-1 [65, 66], which stimulates us to focus on FOXO3a as a downstream target regulated by AMPK and an effective inducer for the autophagy-lysosome system to explore the underlying mechanisms for the prevention and treatment of aging-related atrophy of skeletal muscle during SPD and exercise interventions.

**Figure 9:**
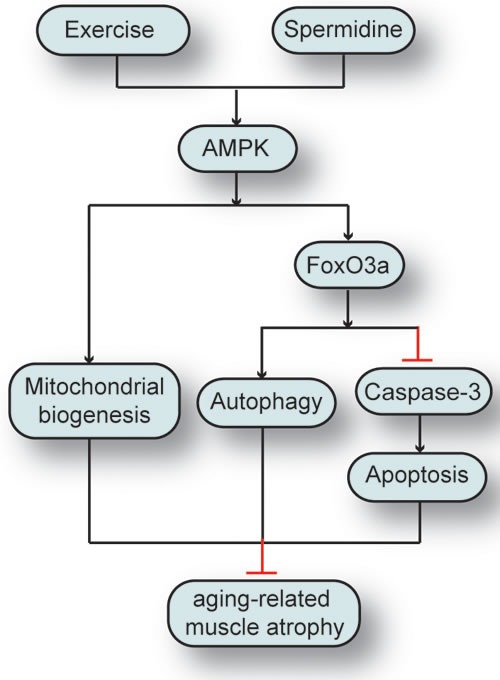
The synergistic effect of spermidine and exercise on the attenuation of aging-related skeletal muscle atrophy through regulating autophagy and apoptosis ***via*** AMPK-FOXO3a signal pathway.

Recently, AMPK has been found to phosphorylate FOXO3a on Ser588 under stimuli triggering, but not to FOXO1 or FOXO4 [67]. As the downstream effector of AMPK signaling, FOXO3a signaling pathway induces the transcriptional activation of atrogenes and autophagy-related genes being in charge of protein degradation. Our study has shown that FOXO3a is necessary for the attenuation of skeletal muscle atrophy induced by D-gal and the maintenance of proliferation in aging skeletal muscle cells by spermidine and exercise. This finding is consistent with its reported role as a downstream target of AMPK. These data suggest that exercise and spermidine intervention for delaying skeletal muscle senescence may share common mediators, and those mediators may act on identical signal pathways at varying degrees to generate the synergistic effect. In addition, physiological dose of D-gal can activate cleaved caspase-3 and suppress AMPK and FOXO3a. Sufficient FOXO3a activity in skeletal muscle may facilitate the effect of exercise and spermidine on cell proliferation. Due to the role of FOXO in mediating NF-κB driven inflammation [68] in skeletal muscle [69], as well as its regulatory role in aging [70] and muscle atrophy [60], the defined role of FOXO3a in senescence programming requires further exploration in skeletal muscle during aging process. Previous studies have documented AMPK activation under the condition of energy stress such as starvation or exercise, thereby leading to the up-regulation of FOXO3a-dependent Atg proteins in primary myotubes, and AMPK-FOXO3a-mediated protein degradation as a source to produce alternative nutrients and energy substrates [71, 72]. It is well studied that the energy-sensing AMPK can directly phosphorylate FOXO3 at three residues in mammalian cells in response to stimuli. AMPK is necessary and sufficient for the phosphorylation of FOXO3a at these sites in cells and animals [67]. Since AMPK-FOXO3a signal pathway is known to be the major mediator of energy metabolism in skeletal muscle, the potential role of AMPK and FOXO3a in autophagic and apoptosis level following exercise and spermidine intervention is also need to be examined. Taken together, these results are consistent with previous studies [13, 20, 21, 55] that exercise and spermidine have multiple protective functions, especially the functions associated with autophagy and apoptosis in vivo. Our results have addressed the functional importance of AMPK and FOXO3a in concert with autophagy and apoptosis regulation to maintain homeostasis of skeletal muscle in the elderly (Figure 9). Furthermore, given the important roles of AMPK-FOXO3a signal pathway in both autophagy and apoptosis, further exploring the contribution of this signal pathway to aging and longevity is highly desired.

In conclusion, regular exercise combined with spermidine intervention may reduce D-gal-induced apoptosis in aging cells through activating autophagy to mitigate the aging process of skeletal muscle. Therefore, combinatorial intervention strategy of exercise and spermidine may be beneficial for aging-related myopathy.

## MATERIALS AND METHODS

### Reagents and antibodies

D-Galactose and spermidine were purchased from Sigma-Aldrich Corporation (St. Louis, MO, USA). Maleic Dialdehyde (MDA) and Superoxide Dismutase (SOD) kits were purchased from Jiancheng Company (Nanjing, China). Primary antibodies such as LC3, Beclin1, p62, Bcl-2, Bax, AMPKα (D5A2), phosphor-AMPKα (Thr72) and FOXO3a were purchased from Cell Signaling Technology (Danvers, MA, USA). The cleaved caspase-3 antibody was obtained from Abcam (Abcam, Cambridge, UK). All secondary antibodies used for Western blot were purchased from Calbiochem (San Diego, CA, USA). Other chemicals were of analytical reagent grade and purchased from Santa Cruz Biotechnology (Santa Cruz, CA, USA).

### Animal grouping and treatments

Four-month-old male Sprague-Dawley (SD) rats (Certificate No. SCXK2015-0018) were purchased from the Experimental Animal Center of Hubei Provincial Disease Control Center (Wuhan, China) and housed at the environment with room temperature of 22 ± 3 °C, relative humidity of 50-70% and light-dark cycle of 12h-12h. The protocols were reviewed and approved by Institutional Animal Care and Use Committee at Wuhan Sports University. Rats were randomly divided into 5 groups depending on treatments including normal control (Con) without treatments, D-gal administration (D), D-gal administration plus spermidine treatment (DS), D-gal administration plus exercise (DE), D-gal administration plus exercise and spermidine (DES) groups with 7 rats in each group.

The rats from D-gal-induced aging groups were subjected to subcutaneous injection of D-gal at the daily dose of 200 mg/kg for 6 weeks. The rats from DS and DES groups were subjected to intraperitoneal injection of sperimidine dissolved in phosphate buffered saline at the daily dose of 5 mg/kg for 6 weeks. The rats from exercise groups (DE and DES groups) were provided with swimming training at the exercise intensity of 45 min/d after 6 h of drug administration during 6-week exercise training period with 5 days training in each week. All rats from Con group were administered with saline at the identical volume for 6 weeks. The body weights of the rats were regularly monitored every week. On the day after the final treatment, all rats were anesthetized with intraperitoneal injection of thiopental sodium and sacrificed for the collection of experimental samples. The laboratory animals were handled in a humane manner in accordance with the Chinese Experimental Animal Administration Legislation.

### Histochemistry staining

After sacrificed, gastrocnemius muscle samples were surgically and immediately removed, washed with physiological saline, embedded in Tissue-Tek OCT compound, followed by snap-frozen in liquid nitrogen and stored at −80 °C until further analysis. The gastrocnemius muscle samples were fixed in 10% neutral-buffered formalin and cut at 5 μm using a cryotome. The sections were dewaxed and rehydrated, and stained for analyzing the damage of gastrocnemius muscle. Masson trichrome staining is used for distinguishing collagen (blue) and skeletal muscle (red). The images of the sections from each group were acquired under a light microscope at a magnification of 200×. Cross-sectional area of selected 150 skeletal muscle fibers in the stained sections was measured and calculated using imageJ software.

### SA-β-gal staining

Senescence-associated β-galactosidase (SA-β-gal) staining was performed using a SA-β-gal staining kit (Genmed Scientifics Inc., Delaware, USA) according to the manufacturer's protocol. The gastrocnemius muscle samples were fixed in 4% (v/v) formaldehyde for 24 h and stained with SA-β-gal staining solution at pH 6.0 for 12 h. The SA-β-gal positive cells exhibited a blue color. The number of positive cells was counted under a phase-contrast microscope.

### TUNEL assay

Deoxynucleotidyl transferase-mediated dUTP nick-end labeling (TUNEL) assay was used to evaluate DNA fragmentation generated in response to apoptotic signals following the method described previously [73]. TUNEL staining was performed using the TUNEL system kit (Roche Applied Science, Indianapolis, USA). In brief, 6-μm thick frozen muscle cross-sections were air dried, and fixed in PBS with 4% paraformaldehyde, pH 7.4, for 20 min at room temperature. The sections were permeabilized with 0.1% Triton X-100 in 0.1% sodium citrate for 2 min at 4 °C, and then incubated with TUNEL reaction mix containing TdT and fluorescein-labeled dUTP for 1 h at 37 °C in a humidified chamber in dark. Subsequently, the section was counterstained with 4’,6’-diamidino-2-phenylindole (DAPI). The number of TUNEL-positive cells per section was counted and calculated as a percentage of all nuclei according to the manufacturer's protocol. Images were recorded under an Olympus fluorescence microscope (Olympus, Tokyo, Japan) at an objective magnification of 800×, with filter blocks for DAPI and fluorescein. For each group in given experiment, at least 300 randomly selected cells were determined for the quantification.

### Immunofluorescence

Skeletal muscle sections (6 μm thick) were stained with anti-cleaved-caspase-3 antibody (Cell Signaling Technology, Danvers, MA, USA). Concentration-matched and species-specific immunoglobulins (Beyotime Biotechnology, Wuhan, China) were used as the control antibodies. After labeling with Cy3-tagged secondary antibody, slices were analyzed under a fluorescence microscope (Olympus, Tokyo, Japan).

### SOD activity and malondialdehyde (MDA) content

SOD activity was determined according to the capability to inhibit the auto-oxidation of pyrogallol. The reaction mixture was composed of 50 mM Tris-HCl, pH 8.2, 1 mM diethylenetriamine penta-acetic acid and the tested sample. The reaction was initiated by the addition of pyrogallol and the absorbance was measured kinetically at 420 nm, 25 °C, for 3 min. One unit of SOD activity is defined as the amount of the sample needed to inhibit pyrogallol oxidation by 50%. The final results were expressed as U/mg protein.

The lipid peroxide was estimated in skeletal muscle using thiobarbituric acid (TBA) reactive substance tests. After the addition of 8.1% sodium dodecyl sulfate, each sample or standard (1,1,3,3-tetraethoxypropane) was vortexed and kept at room temperature for 10 min. At the end of incubation period, 20% acetic acid and 0.6% thiobarbituric acid were added and the test tubes were placed in a water bath at 90-95 °C for 1 h. After that, they were cooled on ice and the colored supernatant was obtained by adding a mixture with butanol-pyridine ratio of 15:1 with sequential vortex and centrifugation. The absorbance was measured at 535 nm. The results were expressed as nmol MDA in each gram of skeletal muscle tissue.

### Western blot

The skeletal muscle samples stored at −80 °C were homogenized in ice-cold RIPA buffer (20 mM Tris, 135 mM NaCl, 2 mM EDTA, 2 mM DTT, 25 mM β-glycerophosphate, 2 mM sodium pyrophosphate, 10% glycerol, 1% Triton X-100, 1 mM sodium orthovanadate, 10 mM NaF, 10 mg/mL aprotinin, 10 mg/mL leupeptin, and 1 mM PMSF, pH 7.5) and centrifuged at 12000 ×g for 30 min at 4 °C. The quantification of protein in supernatant was carried out using a BCA kit (Beyotime Biotechnology, Wuhan, China). Samples were mixed with 5× sample buffer (10% SDS, Tris-HCl 0.5 M (pH 6.8), 25% β-mercaptoethanol, 0.25% bromophenol blue, 50% glycerol) and boiled at 95 °C for 5 min. Proteins (40 μg for each lane) were separated on 12% SDS-polyacrylamide gel and transferred to a nitrocellulose membrane by electrophoretic transferring. Blocking was carried out with 5% BSA in TBS (10 mM Tris, pH 7.0, 150 mM NaCl) containing 0.1% (v/v) Tween-20 (TBS-T) for 1 h at room temperature. The immobilized proteins were incubated with anti-LC3 and anti-p62 antibody (1:1000, Cell Signaling Technology, Danvers, MA, USA) overnight at 4 °C. After being washed for 3 × 15 min in TBS-T buffer, the membrane was incubated with goat horseradish peroxidase (HRP)-conjugated goat anti-rabbit IgG (1:20000, Santa Cruz Biotechnology, Santa Cruz, CA, USA) at room temperature for 1 h. The membrane was washed three times in TBS-T buffer as described above. The protein bands were visualized using chemiluminescent HRP substrates (Millipore, Billerica, MA, USA). The target protein was probed by corresponding antibody, and then visualized by enhanced chemiluminescence (ECL) reagent imaged by ultra-sensitive fluorescence/chemiluminescence imaging system ChemiScope6300 (CLiNX Science Instruments, Shanghai, China).

### Transmission electron microscopic examination

The samples of gastrocnemius muscle with the volume of 1 mm^3^ were removed and fixed in 2.5% glutaraldehyde in phosphate buffer for 2 h, and then rinsed with 1 mmol/L phosphoric acid solution and fixed in 1% osmium tetroxide for 2-3 h. The blocks were cut carefully into ultrathin (0.07 μm) sections. The sections were stained with 3% uranylacetate and lead citrate and then viewed under a JEOL JEM1400 transmission electron microscope (Peabody, MA, USA) at Wuhan Institute of Virology, Chinese Academy of Sciences.

### Statistical analysis

All data were expressed as mean ± standard error (M ± SE). The significant difference between groups was validated with one-way ANOVA using GraphPad Prism 6 (GraphPad Software Inc., La Jolla, CA, USA). The significant difference was considered at *P* < 0.05.
